# A Novel Solid-Phase Site-Specific PEGylation Enhances the *In Vitro* and *In Vivo* Biostabilty of Recombinant Human Keratinocyte Growth Factor 1

**DOI:** 10.1371/journal.pone.0036423

**Published:** 2012-05-04

**Authors:** Zhifeng Huang, Guanghui Zhu, Chuanchuan Sun, Jingui Zhang, Yi Zhang, Youting Zhang, Chaohui Ye, Xiaojie Wang, Dariush Ilghari, Xiaokun Li

**Affiliations:** 1 Key Laboratory of Biotechnology and Pharmaceutical Engineering of Zhejiang Province, Wenzhou Medical College, Wenzhou, China; 2 The 2nd Affiliated Hospital Medical Center, Wenzhou Medical College, Wenzhou, China; 3 Department of Pharmacology, New York University School of Medicine, New York, New York, United States of America; 4 Normal Bethune Medical College, Jilin University, Changchun, China; University of Hong Kong, China

## Abstract

Keratinocyte growth factor 1 (KGF-1) has proven useful in the treatment of pathologies associated with dermal adnexae, liver, lung, and the gastrointestinal tract diseases. However, poor stability and short plasma half-life of the protein have restricted its therapeutic applications. While it is possible to improve the stability and extend the circulating half-life of recombinant human KGF-1 (rhKGF-1) using solution-phase PEGylation, such preparations have heterogeneous structures and often low specific activities due to multiple and/or uncontrolled PEGylation. In the present study, a novel solid-phase PEGylation strategy was employed to produce homogenous mono-PEGylated rhKGF-1. RhKGF-1 protein was immobilized on a Heparin-Sepharose column and then a site-selective PEGylation reaction was carried out by a reductive alkylation at the N-terminal amino acid of the protein. The mono-PEGylated rhKGF-1, which accounted for over 40% of the total rhKGF-1 used in the PEGylation reaction, was purified to homogeneity by SP Sepharose ion-exchange chromatography. Our biophysical and biochemical studies demonstrated that the solid-phase PEGylation significantly enhanced the *in vitro* and *in vivo* biostability without affecting the over all structure of the protein. Furthermore, pharmacokinetic analysis showed that modified rhKGF-1 had considerably longer plasma half-life than its intact counterpart. Our cell-based analysis showed that, similar to rhKGF-1, PEGylated rhKGF-1 induced proliferation in NIH 3T3 cells through the activation of MAPK/Erk pathway. Notably, PEGylated rhKGF-1 exhibited a greater hepatoprotection against CCl_4_-induced injury in rats compared to rhKGF-1.

## Introduction

Keratinocyte growth factor 1 (KGF-1), also known as fibroblast growth factor 7 (FGF-7), is a paracrine-acting mitogen produced by cells of mesenchymal origin in response to pro-inflammatory cytokines and steroid hormones [1], [2], [3], [4]. KGF-1 is a heparin binding growth factor that acts exclusively through a splicing variant of FGF receptor 2, FGFR2-IIIb, which is expressed predominantly by epithelial cells in the tongue, oral mucosa and gastrointestinal tract as well as liver, lung and pancreas [5]. Functional assays in organ and cell cultures and *in vivo* analysis show that KGF-1 is mitogenically active only on epithelial cells derived from a variety of tissues [6], [7] without any tumorigenic effect. Since KGF-1 impacts proliferation and differentiation in parenchymal epithelial cells of differentiated tissues, it has been proposed for treatment of pathologies associated with dermal adnexae, liver, lung, and the gastrointestinal tract diseases, particularly wound healing in various tissues and organs [8], [9]. Recombinant human KGF-1 (rhKGF-1) can significantly reduce the duration and incidence of oral mucositis after intensive chemotherapy and radiotherapy and autologous hematopoietic stem-cell transplantation. RhKGF-1, therefore, seems a major breakthrough in the management of patients receiving intensive treatment for solid tumours and haematological malignancies. This has led the Food and Drug Administration (FDA) to approve rhKGF-1, also known as Palifermin, for the prevention of oral mucositis of patients with haematological malignancies and boosted the development of other mucosa-protective growth factors, such as repifermin (KGF-2) and velafermin (FGF-20).

Therapeutic applications of rhKGF-1 have been restricted by its low level of expression in various expression systems, poor stability and a relatively short half-life *in vivo* [10]. The protein unfolds around body temperature (∼37°C) and the unfolded protein aggregates rapidly [11], [12]. Moreover, frequent administration (daily) of rhKGF-1 often results in impaired homeostasis *in vivo* and may cause severe adverse effects such as rash, erythema, edema, pruritus, dysesthesia, mouth/tongue thickness/discoloration, and taste alteration [9]. Given these limiting factors, high priority should be given to the development of strategies that could significantly enhance the stability and circulating half-life of rhKGF-1.

Approaches to improve the stability and pharmacokinetic properties of therapeutic proteins include structural modification and covalent conjugation of polyethylene glycol (PEGylation) [13], [14]. PEGylation of drugs and therapeutic proteins has become one of the most mature, valid and widely used drug delivery technologies ever developed. It has been previously reported that PEG-conjugated therapeutic peptides/proteins exhibit clinical properties superior to those of their corresponding unmodified parental molecules [15], [16], [17]. PEGylation delays drug absorption thereby reducing serious side-effects caused by acute peak drug concentration, and also prolongs the half-life of drugs and therefore improves patients' compliance by greatly reducing the frequency of drug injections [15]. PEGylation is a reaction between functional groups of an activated PEG and those of specific residues, such as lysine, cysteine and N-terminal residues, in a protein structure [18]. Presence of several reactive amino acids on a protein surface results in random non-site-specific mono- and multiple-PEGylations, which in turn may mask or interfere with receptor binding sites of PEGylated proteins and may also lead to a dramatic reduction in their biological activities [19]. Hence, a site-specific and well-controlled PEGylation might be the attractive possibilities offering the advantages to retain bioactivity of therapeutic proteins and to generate chemically identical entities with predictable pharmaceutical behavior.

In the present study, for the first time, we propose solid-phase PEGylation for a site-specific modification of rhKGF-1. Using this novel PEGylation approach, we successfully modified the rhKGF-1 at the N-terminal residue with 20 kDa mPEG-butyraldehyde and produced a highly homogeneous mono-PEGylated rhKGF-1. Our bioactivity and CD analysis data showed that solid-phase PEGylation did not impinge on the activity and structure of rhKGF-1. Furthermore, our *in vitro* and *in vivo* biostability analyses showed that PEGylated rhKGF-1 was significantly more stable than non-PEGylated rhKGF-1. Importantly, PEGylated rhKGF-1 was found to be significantly more effective than rhKGF-1 in protecting liver against CCl_4_-induced injury in rats.

## Results

### Preparation of rhKGF-1

**Figure 1:**
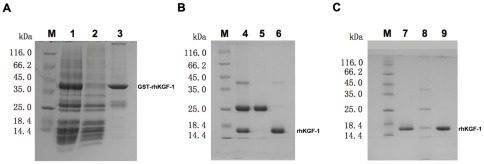
SDS-PAGE analysis of expression and purification of rhKGF-1. Panel A. SDS-PAGE analysis of the fractions collected from Glutathione Sepharose chromatography. Lane M, molecular weight standards; lane 1, the cell lysate supernatant of BL21 (DE3) containing pET- GST-KGF-1 induced with 1.0 mM IPTG for 4 h; lane 2, the flow-through fraction; lane 3, the fraction eluted with 50 mM Tris-HCl, 10 mM reduced glutathione, pH 8.0. Panel B. SDS-PAGE analysis of the fractions collected from CM Sepharose chromatography. Lane M, molecular weight standards; lane 4, the GST-rhKGF-1 fusion protein after treatment with thrombin; lane 5, the flow-through fraction; lane 6, NaCl-eluted fraction. Panel C. SDS-PAGE analysis of Heparin Sepharose chromatography. Lane M, molecular weight standards; lane 7, NaCl-eluted fraction from CM Sepharose Fast Flow column; lane 8, 0.45 M NaCl-eluted fraction; lane 8, 0.6 M NaCl-eluted fraction.

The cDNA of rhKGF-1 was cloned into the *Nde*I and *Bam*HI restriction sites of pET-GST and then the recombinant vector was used to transform bacterial host BL21(DE3). The transformants bearing the expression vector were induced with IPTG and then lyzed, and the soluble fraction was collected following centrifugation and filtration. SDS-PAGE analysis of the lysate supernatant showed that the recombinant protein was expressed as a soluble product with an apparent molecular weight of about 38 kDa corresponding to the predicted protein product of GST-KGF-1 (**Figure 1A**). The soluble product was then purified as described in *Materials and Methods*. Next, the N-terminal GST tag was cleaved from GST-rhKGF-1 by digestion with thrombin according to the manufacturer's instruction. Residual GST that survived enzymatic treatment together with the remaining uncleaved GST-rhKGF-1 were then removed from rhKGF-1 preparation by a two step chromatography using CM Sepharose Fast Flow and heparin–Sepharose columns. As demonstrated in **Figure 1B and 1C**, SDS-PAGE and scanning densitometry analysis showed that the recovered untagged rhKGF-1 was homogenous and its purity is over 95%.

### Preparation of Solid-Phase Mono-PEGylated rhKGF-1

**Figure 2:**
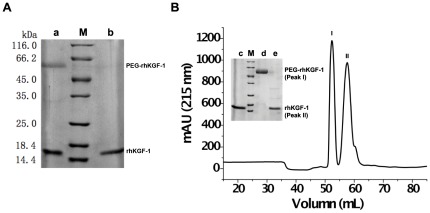
SDS-PAGE analysis and elution profile of solid-phase PEGylation reaction mixture. Panel A. SDS-PAGE analysis of solid-phase PEGylated rhKGF-1. Lane M, molecular weight standards; lane a, solid-phase PEGylation products obtained at PEG-to-protein ratio of 10/1 and reaction time of 8 h; lane b, non-PEGylated rhKGF-1. Panel B. Elution profile of PEGylation reaction mixture on a SP Sepharose Fast Flow column. The Insert panel shows SDS-PAGE analysis of the fractions collected from SP Sepharose chromatography.

Given the fact that rhKGF-1 has a high affinity for heparin, the recombinant protein was first adsorbed to a heparin affinity column. Next, mPEG20 kDa-butyraldehyde (Alk-mPEG) was introduced to a reducing agent and then the mixture was circulated through the heparin column at very low flow rate. Upon circulation of Alk-mPEG through the column, Alk-mPEG covalently attached to N-terminal residue (α-amine) of rhKGF-1 via a reductive alkylation. To determine the optimal conditions for the site-specific PEGylation of the recombinant protein, the effects of different PEG/protein molar ratios (ranging from 5/1 to 30/1) and reaction times (ranging from 2 to 12 hours) on the yield and efficiency of PEGylation were examined. Our data showed that the optimized yield of mono-PEGylation was achieved when the reaction was performed at pH 6 for 8 hours using a PEG/protein molar ratio of 10/1 (**Figure 2A**). In fact, our SDS-PAGE and scanning densitometry analysis showed that when the optimal condition was provided, over 40% (i.e. 42.3%) of the rhKGF-1 used in the reaction was successfully mono-PEGylated.

To separate PEGylated rhKGF-1 from non-PEGylated rhKGF-1, the bound materials, i.e. both PEGylated and non-PEGylated rhKGF-1, on the heparin column was eluted using a high salt sodium phosphate buffer and then the eluted protein sample was applied on a Sepharose G25 column to be desalted. The desalted protein sample was then subjected to a salt gradient cation-exchange chromatography on a HiTrap SP Sepharose Fast Flow column. After the cation-exchange chromatography, PEGylated and non-PEGylated rhKGF-1 were eluted in two separate peaks as confirmed by SDS-PAGE analysis (**Figure 2B**), suggesting that the modification alters the electrostatic properties of rhKGF-1 surface.

### Validation of Solid-Phase PEGylated rhKGF-1

**Figure 3:**
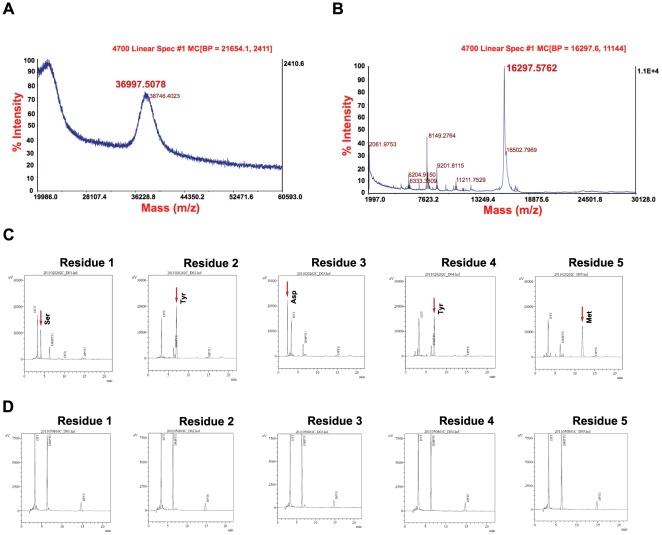
Validation of the solid-phase PEGylated rhKGF-1. Panel A, MALDI-TOF mass spectrometry of PEGylated rhKGF-1 showing the molecular mass of PEGylated rhKGF-1 (36997 Da). Panel B, MALDI-TOF mass spectrometry of non-PEGylated rhKGF-1 showing the molecular mass of non-PEGylated rhKGF-1 (16297 Da). Panel C, D. N-terminal sequencing of non-PEGylated and PEGylated rhKGF-1 by Edman degradation method.

To confirm that rhKGF-1 was mono-PEGylated, MALDI-TOF mass spectrometry (MALDI-TOF-MS) was employed. Our MS data showed that the PEGylated rhKGF-1 had a molecular weight of 36997.50 Da (**Figure 3A**), therefore confirmed that a single 20 kD PEG molecule was conjugated to rhKGF-1 (16297.57 Da) (**Figure 3B**). The broad MS peak corresponding to PEGylated rhKGF-1 was most likely due to the PEG polydispersity as reported before [20]. Next, an automated N-terminal sequencing by Edman degradation was employed to validate the site of PEGylation on rhKGF-1. Using this method of sequencing, the five N-terminal amino acids (Ser-Tyr-Asp-Tyr-Met) were detected only in the non-PEGlyated rhKGF-1 (**Figure 3C**) but not in the PEGylated rhKGF-1 (**Figure 3D**). The N-terminal residues of PEGylated rhKGF-1 were not detected because the covalent attachment of PEG to the N-terminal α-amino group did not allow the N-terminal Ser residue to be modified by 1-fluoro-2,4-dinitrobenzene(FDNB) through the sequencing reaction therefore made it unrecoverable [21]. Taken together, these sequencing data unambiguously corroborated site-specific N-terminal PEGylation of rhKGF-1.

### Analysis of Bioactivity of Solid-Phase PEGylated rhKGF-1

**Figure 4:**
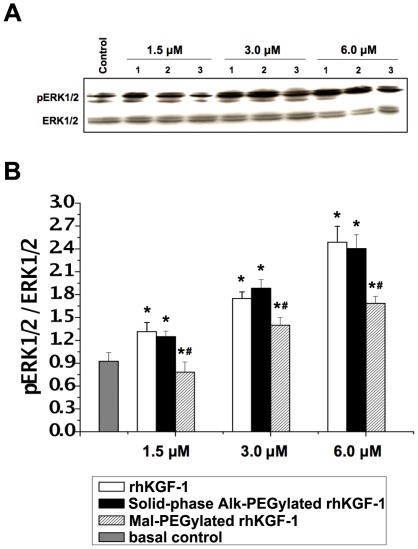
The effect of rhKGF-1 PEGylation on MAP kinase activation. Panel A. NIH 3T3 fibroblasts were stimulated with rhKGF-1 or PEGylated rhKGF-1 at three does 1.5 µM, 3 µM, and 6 µM, and phosphorylation courses of ERK1/2 (p-ERK) were measured by immunoblotting alongside total ERK as loading controls. The blots shown are representative of three independent experiments; Panel B. Semi-quantitative analysis of the protein bands in Panel A. *, p<0.05 *vs* Normal control; ^#^, p<0.05 *vs* the corresponding non-PEGylated rhKGF-1 group.

To evaluate the bioacitivity of PEGylated rhKGF-1, proliferative effect of PEGylated rhKGF-1 on NIH 3T3 cells was compared to that of non-PEGylated rhKGF-1 using standard MTT assay. As shown in **Table 1**, non-PEGylated rhKGF-1 and PEGylated rhKGF-1 induced comparable mitogenic response in NIH 3T3 cells, demonstrating that the mono-PEGylation had no adverse effect on proliferative activity of rhKGF-1. Furthermore, we qualitatively analyzed the effect of rhKGF-1 PEGylation on the KGF-1-induced activation of MAP kinase pathway, a key KGF-1 signaling pathway that is involved in cell proliferation, in NIH 3T3 cells. As shown in **Figure 4**, similar to rhKGF-1, PEGylated rhKGF-1 triggered intense phosphorylation of Erk1 and Erk2, further confirming that the solid-phase PEGylation did not impact on the bioactivity of rhKGF-1.

**Table 1:** Biological activity of native rhKGF-1 and PEGylated rhKGF-1.

	rhKGF-1	Alk-PEGylated rhKGF-1 by solid-phase	Mal-PEGylated rhKGF-1 by solution-phase
**Mean biological Activity (IU/µmol)**	2.21×10^5^	1.98×10^5^	3.82×10^4^
**Relative activity (%)**	100%	89.6%	17.3%

### Effect of Solid-Phase PEGylation on the Structural Integrity and Stability of rhKGF-1

**Figure 5:**
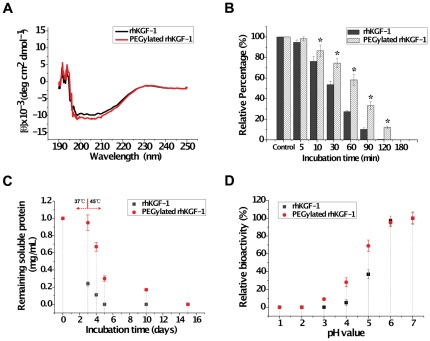
The effect of solid-phase PEGylation on the structural integrity and stability of rhKGF-1. Panel A. Far-UV CD spectra of non-PEGylated (black line) and solid-phase PEGylated rhKGF-1 (red line). The ellipticities are reported as mean residue ellipticity (MRE) (deg cm^2^ dmol^−1^). Panel B. Effect of PEGyaltion on the proteolytic stability of rhKGF-1. PEGylated and intact rhKGF-1 were incubated with trypsin with a molar ratio of 10/1 (protein/trypsin) at 37°C for the indicated periods of times. Trypsin-treated rhKGF-1 was examined for the protein integrity by SDS-PAGE. The bands representing PEGylated and intact rhKGF-1 were quantified by densitometry scanning. The band densities of non-trypsin-treated intact and PEGylated rhKGF-1 are considered as 100% (indicated as control), while the band densities of trypsin-treated PEGylated or intact rhKGF-1 are presented as relative percentage to the control. *, p<0.05 *vs* the corresponding intact rhKGF-1 group. Panel C. Kinetic plots of remaining soluble non-PEGylated rhKGF-1 (black square) and PEGylated rhKGF-1 (red circle). The soluble protein concentration was determined by Bicinchoninic acid (BCA) kit. Samples were first incubated at 37°C for 3 days and then transferred to 45°C for the rest of the experiments. Pane D. Effect of pH on *in vitro* bioactivity of non-PEGylated rhKGF-1 (black square) and PEGylated rhKGF-1 (red circle).

To investigate whether the solid-phase PEGlyation affects the rhKGF-1 structure, the secondary structure of PEGylated rhKGF-1 was compared to that of non-PEGylated rhKGF-1 using circular dichroism (CD) spectroscopy. Far-UV CD spectra recorded for PEGylated rhKGF-1 was comparable to that of non-PEGylated rhKGF-1 (**Figure 5A**), demonstrating that the solid-phase PEGylation of rhKGF-1 did not alter the secondary structure of rhKGF-1.

Therapeutic proteins are exposed to a variety of stresses that can result in protein unfolding or degradation. Using a rational stabilization method, the biopharmaceutical proteins can be modified such that their structures and activities are substantially more robust with respect to protease exposures, changes in temperature and pH [19], [22]. PEGylation, as a simple strategy for protein stabilization, would be expected to increase proteolytic resistance of rhKGF-1. To test this, the stability of rhKGF-1 and its mono-PEGylated derivative in the presence of trypsin were investigated *in vitro*. We selected trypsin because this proteolytic enzyme has been extensively used to investigate resistance of a protein to enzymatic digestion [23], [24]. As shown in **Figure 5B**, there was significant difference in the amount of degradation between non-PEGylated and PEGylated rhKGF-1 incubated with trypsin for various time periods. After incubation with trypsin for 60 min at 37°C, nearly 58.2% of PEGylated rhKGF-1 still remained intact, whereas only about 27.4% non-PEGylated rhKGF-1 was left intact, indicating that solid-phase PEGylation significantly increased resistance of rhKGF-1 to proteolysis.

Protein aggregation is another major stability problem of therapeutic proteins. To check whether PEGylation can improve the solubility of rhKGF-1, the aggregation tendency of PEGylated rhKGF-1 was compared to that of rhKGF-1 by measuring the amount of the protein aggregates accumulated at elevated temperatures over a long period of time. As shown in **Figure 5C**, the PEGylation showed pronounced stabilizing effect on rhKGF-1 by preventing aggregation. At 37°C, the rhKGF-1 sample had already lost about 76% of the soluble rhKGF-1 over three days, while the PEGylated sample showed almost no loss. At 45°C, the rhKGF-1 sample almost rapidly lost all the remaining soluble rhKGF-1, whereas the PEGylated sample showed much slower loss rate of the soluble PEGylated rhKGF-1. This comparative analysis, therefore, demonstrated that PEGylation significantly suppressed tendency of rhKGF-1 for aggregation.

It is known that rhKGF-1 rapidly inactivated at acidic conditions [22]. To test whether PEGylation can protect rhKGF-1 against low pH-induced inactivation, using a mitogenic bioassay, the bioactivity of PEGylated rhKGF-1 was compared to that of rhKGF-1 within a pH range between 1.5 to 7 (**Figure 5D**). The bioactivities were normalized to their values at neutral pH (pH 7.0). The mitogenic bioassay data demonstrated that the *in vitro* bioactivity of both rhKGF-1 and its PEGylated derivative declined as pH decreased. However, compared to rhKGF-1 that lost 63% of its bioactivity at pH 5.0, PEGylated rhKGF-1 showed about 25% loss in its bioactivity. When the pH value decreased to 4.0, rhKGF-1 almost lost all of its bioactivity, whereas solid-phase PEGylated rhKGF-1 still retained 28% of its bioacitivity. These bioassay analyses, therefore, confirmed that PEGylation could improve biostability of rhKGF-1 in acidic pH.

### Pharmacokinetic Study of Solid-Phase PEGylated rhKGF-1 in Rats

**Figure 6:**
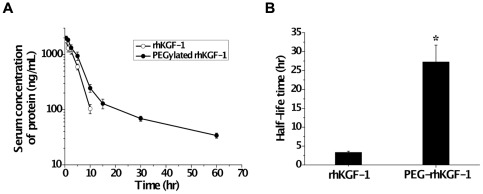
Pharmacokinetics study of solid-phase PEGylated rhKGF-1 in rats. Panel A. Normal male SD rats were injected intravenously with 100 µg/kg rhKGF-1 (open circle), and PEGylated rhKGF-1 (solid circle). Blood samples were collected at the indicated time points. The amount of rhKGF-1 was measured by Human KGF-basic Mini ELISA Development Kit. A standard curve was made for each rhKGF-1, n = 6. Values are the mean±SD. Panel B. Comparison of half-life of non-PEGylated rhKGF-1 and PEGylated rhKGF-1. *, p<0.01 *vs* the corresponding non-PEGylated rhKGF-1 group.

The *in vivo* half-life times of the two forms of rhKGF-1 were analyzed by intravenously (i.v.) injecting a single dose of 100 µg/kg of intact or PEGylated rhKGF-1 in male SD rats and measurements of the dynamic levels of two proteins in the blood using the KGF (FGF-7)-basic Mini ELISA Development Kit. Based on the pharmacokinetic curves shown in **Figure 6A**, the plasma elimination half-life (t_1/2_) was calculated with Drug and Statistics Software (DAS, Version. 2.0; Mathematical Pharmacology Professional Committee of China) and the equation described in the *Materials and Methods*. Consistent with previously published reports [25], [26], our pharmacokinetic data showed that the non-PEGylated rhKGF-1 was eliminated with a plasma half-life (t_1/2_) of about 3.22 h. Importantly, PEGylation increased the plasma half-life of rhKGF-1 to about 27.26 h, an 8.2-fold increase compared to intact rhKGF-1 (**Figure 6B**).

### Comparison of Hepatoprotective Effects of Solid-Phase PEGylated rhKGF-1 and Intact rhKGF-1 Using a Rat Model of Acute Liver Failure

**Figure 7:**
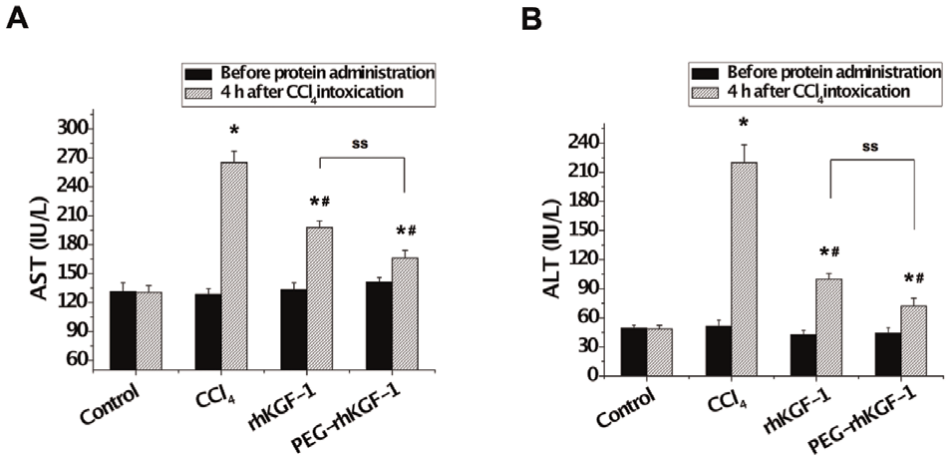
Effect of solid-phase PEGylated rhKGF-1 on serum AST and ALT levels in rats intoxicated with CCl_4_. 48 male SD rats were randomly divided into four groups (12 rats/group): enalpril interventional group A (rhKGF-1-pretreated groups), enalpril interventional group B (PEGylated rhKGF-1-pretreated groups), injury-model group and healthy control group. The test groups were pretreated with non-PEGylated and PEGylated rhKGF-1 24 hours prior to 2.5 mL/kg CCl_4_ administration. Four hours after the intraperitoneal injection of CCl_4_, rats were killed and their serum AST/ALT levels were determined. *, p<0.05 *vs* control group; ^#^, p<0.05 *vs* injury-model group; ^ss^, p<0.05 between indicated groups.

Our biochemical analyses clearly demonstrated that PEGylated rhKGF-1 has a similar *in vitro* bioactivity, but significantly higher *in vitro* and *in vivo* biostability compared to its intact counterpart. We next compared the therapeutic effect of PEGylated rhKGF-1 to that of intact rhKGF-1 using a rat model of acute hepatic failure. To this end, two groups of rats (n = 12) were pre-treated with a single dose (0.1 µmol/kg) of either rhKGF-1 or PEGylated rhKGF-1, and 24 hours later they were intoxicated once by CCl_4_ (2.5 mL/kg). The hepatoprotective effects of rhKGF-1 and PEGylated rhKGF-1 were then studied by comparing the serum levels of aspartate aminotransferase (AST) and alanine aminotransferase (ALT) just before the rhKGF-1/PEGylated rhKGF-1 injection and 4 hours after the CCl_4_ administration. As a control group, 12 rats were only treated with normal saline. Additionally, an injury- model group (n = 12) was pretreated with normal saline prior to intoxication by CCl_4_. As shown in **Figure 7**, compared to the healthy control group in which the serum levels of AST and ALT remained unchanged during the course of experiments, the serum levels of the aminotransferases (AST and ALT) in the injury-model group were significantly increased (2-fold and 4.5-fold, respectively) above the baseline levels after CCl_4_ administration. As expected, that the pretreatment of the test groups with either intact rhKGF-1 or PEGylated rhKGF-1 considerably protected liver against injury in the CCl_4_-intoxicated animals (**Figure 7**). Importantly, PEGylated rhKGF-1 afforded a better hepatoprotection compared to intact rhKGF-1. This is evidenced by the fact that following CCl_4_ intoxication, the serum level of AST and ALT were elevated 1.5-fold and 2.5-fold, respectively, above baseline levels in the rats pretreated with rhKGF-1, whereas the enzyme levels were raised 1.1-fold and 1.5-fold, respectively, above baselines in the serum of animals pretreated with PEGylated rhKGF-1 (**Figure 7**).

## Discussion

A wealth of pharmacological studies has been previously shown that KGF-1, a member of the FGF family, has a high therapeutic potential in preventing oral mucositis in patients with haematological malignancies [8], [26]. In addition, several *in vivo* studies using different animal models have shown that recombinant human KGF-1 could enhance the regenerative capacity of epithelial tissues and protect them from a variety of toxic exposures [6], [7]. However, similar to other therapeutic proteins, the inherent sensitivities associated with rhKGF-1, including thermal instability, degradation by proteolysis, and short half-life *in vivo*, have restricted its clinical applications [10], [22]. A widely used method to evade these limitations is the covalent attachment of PEG molecular to a protein using a solution-phase batch process, a technique is known as solution-phase PEGylation. This approach employs compounds that couple PEG to free amines, typically at lysine residues or at the N-terminal amino acid [27], [28]. A critical limitation of this method is that proteins typically contain several lysine residues, in addition to the N-terminal amino acid. The PEG moiety can couple to the protein at any of the available free amines, leading to a heterogeneous product mixture consisting of mono-, di-, tri-, etc, PEGylated species [19], [28]. The different PEGylated species often have different intrinsic biological activities [29], [30]. This can restrict development of any therapeutic protein including rhKGF-1 because predictability of biological activity and manufacturing reproducibility are essential for regulatory approval. In addition, multiple steps of purification are required to remove undesired impurities, such as the reducing agent, unreacted PEG, and multi-PEGylated species.

It has been previously shown that site-specific PEGylation can overcome the problems of product heterogeneity caused by the solution-phase amine-PEGylation [13], [14]. Site-specific PEGylation can be achieved by the covalent attachment of cysteine-reactive PEG molecule (mPEG-maleimide, Mal-mPEG) to the thiol group of a “free" cysteine residue, i.e., a cysteine residue not involved in a disulfide bond, in a protein structure [28], [31]. The primary structure of rhKGF-1 produced in our laboratory contains five cysteine residues located at positions 1, 15, 40, 102 and 106. Previous studies have shown two disulfide-bonds between Cys 1 and 15, and between Cys 102 and 106 in the structure of rhKGF-1 [22], thus suggesting that the thiol group of Cys 40 is free available for site-specific PEGylation. Since we failed in our attempts to couple this free cysteine (Cys 40) with Mal-mPEG, we decided to conjugate Mal-mPEG with rhKGF-1 at Cys 40 in the presence of chaotrope, i.e. urea. Urea, as a chaotrope, can disrupt the protein–protein and protein–water contacts and interacts preferentially with the protein molecule [32], perhaps exposing Cys 40 to the solvent environment. Although the site-specific PEGylation in the presence of 2 M urea was successful (**Figure S1A**), this modification had detrimental effects on the bioactivity (**Table 1**) and structure (**Figure S1B**) of rhKGF-1. This is most likely because Cys 40 is buried in the hydrophobic core of rhKGF-1 (**Figure S1C**) [22] and plays an important role in maintaining the structural integrity of the protein.

Having failed to produce a bioactive cysteine-PEGylated rhKGF-1, we decided to selectively PEGylated the N-terminal residue of the protein by a reductive alkylation at low pH in solution phase. Under such reaction conditions, PEGylation at the N-terminal amino group (pKa 7.6 to 8.0) is favored over the ε-amino groups of lysine residues (pKa 10.0 to 10.2). This approach has been previously utilized to PEGylate epidermal growth factor (EGF) [30], recombinant human endostatin [33], interferon β-1b [34] and rhFGF21 [20]. N-terminal site-specific PEGylated proteins are generally synthesized by solution-phase chemical reactions where reactants, product, and byproduct remain within the same homogeneous reaction mixture until separation. Therefore, even when reductive alkylation at low pH is carried out, synthesis of some undesirable PEGylated forms is inevitable. As shown in **Figure S2**, under optimal conditions of PEG-to-protein molar ratio (5∶1), reaction time (8 hours) and pH (pH 6), the reductive alkylation (PEGylation) of amino groups in rhKGF-1 yielded both the mono-PEGylated (46.1% of the total protein) and di-PEGylated (11.4% of the total protein) forms of the protein.

Given separation of the mono- and di-PEGylated species of rhKGF-1 was practically unfeasible by typical heparin affinity, size exclusion and ion exchange chromatography methods (data not shown), we used a novel solid-phase PEGylation approach to generate homogenously N-terminal mono-PEGylated rhKGF-1. In this approach, we immobilized rhKGF-1 on a heparin-sepharose column and then passed activated Alk-mPEG through the column. As confirmed by our densitometry, mass spectrometry and sequencing analyses, under optimal conditions, over 40% of rhKGF-1 utilized in the PEGylation reaction is homogenously mono-PEGylated at its N-terminal residue. We have previously shown that the solution-phase PEGylation alters heparin-binding ability of rhKGF-2 [35]. Our salt gradient heparin-sepharose affinity chromatography has shown that the modified rhKGF-2 is eluted at lower salt concentration compared to the intact rhKGF-2, indicating that the solution-phase PEGylation reduces the heparin-binding affinity of rhKGF-2 [35]. The solid-phase PEGylation method reported here, on the contrary, enables us to orientate the protein so that its heparin- and most likely receptor-binding sites are held towards the solid-phase interface, thus allowing us to restrict conjugations at the sites that interfere with the activity of rhKGF-1. This is confirmed by our heparin-sepharose affinity chromatography data demonstrating that PEGylated rhKGF-1 and intact rhKGF-1 bind with a similar affinity to the heparin-sepharose column and co-elute from the column using a linear salt gradient (**Figure S3**). Furthermore, our bioactivity data show that, similar to rhKGF-1, solid-phase PEGylated rhKGF-1 is able to induce proliferatation through the activation of MAPK/Erk pathway in NIH 3T3 cells, thus suggesting that solid-phase PEGylation does not adversely affect the heparin- and receptor-binding ability of rhKGF-1. This conclusion is also supported by our CD analysis showing that solid-phase PEGylated rhKGF-1 and rhKGF-1 have similar secondary structures (**Figure 5A**).

PEGylation may affect physicochemical and/or pharmacokinetic features of the proteins by steric hindrance and conformational changes [19], [20]. Here we demonstrate that the solid-phase PEGylation increases the resistance of rhKGF-1 to trypsin treatment (**Figure 5B**), and to changes in temperature and pH (**Figure 5C and D**), compared to non-PEGylated rhKGF-1. The increased resistance to proteolytic degradation presumably reflects the steric hindrance of the PEG strands that act as direct barriers to cleavage. The aggregation observed at the elevated temperatures (37°C, and 45°C) may be explained by reduced thermodynamic stability of the protein structure and an increased hydrophobic interaction from exposed hydrophobic patches. The conjugated PEG might swim around the hydrophobic patches of rhKGF-1 to suppress hydrophobic interactions. This hydrophobic mechanism might be similar to hydrophobic interactions that suppress the aggregation during the process of in vitro refolding [36]. Taken together, our stability analyses suggest that Alk-mPEG modifies rhKGF-1 such that its structure and activity are substantially more robust with respect to stresses that can cause protein unfolding or degradation.

From our pharmacokinetic data, it appears that the solid-phase PEGylation improves the plasma half-life of rhKGF-1 to about 28 hours, which is over eight-times longer than that of intact rhKGF-1 (**Figure 6**). Mono-PEGylation increases the circulating half-life of rhKGF-1 most likely by reducing proteolysis, and restricting tissue-distribution such as glomerular filtration by the kidney and hepatic uptake. This effect is attributed to the increased molecular size and steric hindrance that result from attaching PEG to the protein [37].

Another important finding of the present study is physiological confirmation that the solid-phase PEGylated rhKGF-1 is more effective than intact rhKGF-1 in protecting liver against CCl_4_-induced injury in rats. This is evidenced by the fact that serum levels of the liver injury biomarkers, ALT and AST, in the CCl_4_-intoxicated rats pretreated with rhKGF-1 were significantly higher than in the animals pretreated with PEGylated rhKGF-1 (**Figure 7**). FGFs, including KGF-1 regulate liver development [38], [39], and regeneration [40], [41], therefore potentially provide a line of defense against CCl_4_-induced hepatocellular necrosis. Moreover, Bohm et al., [42] have previously shown that KGF-1 activates hepatocyte FGFR2-IIIb, reducing CCl_4_-induced centrilobular necrosis and inflammation. These authors have suggested that KGF-1 highly up-regulates expressions of two transcriptional regulators, Dbp and Tef, thereby increasing expression of detoxifying cytochrome P450 enzymes in mouse hepatocytes. This increase in expression of genes that control detoxification, could additionally provide a potential mechanism by which KGF detoxifies CCl_4_ and protects rats from CCl_4_-induced hepatic damage. The increased *in vivo* biostability of PEGylated rhKGF-1 relative to intact rhKGF-1 may explain the observed higher hepatoprotection.

In summary, in order to increase rhKGF-1's stability, half-life and therapeutic potency, we N-terminally mono-PEGylated rhKGF-1 for the first time using a novel “on column" (Heparin-sepharose column) PEGylation method. The PEGylated rhKGF-1 was purified to homogeneity using a cation-exchange chromatography. Similar to the intact rhKGF-1, PEGylated rhKGF-1 induced proliferation via the activation of MAPK/Erk pathway in NIH 3T3 cells. The solid-phase PEGylation remarkably increased the *in vivo* and *in vitro* stability without altering the over all structure and compromising the heparin- and receptor-binding ability of rhKGF-1. The solid-phase PEGylation also considerably improved plasma half-life of rhKGF-1 *in vivo*. Importantly, PEGylated rhKGF-1 afforded a greater hepatoprotection against CCl_4_-induced liver damage in rats compared to rhKGF-1. Collectively, using the sold-phase PEGylation approach reported here, we developed a rhKGF-1 with enhanced stability and an extended plasma half-life for therapeutic applications.

## Materials and Methods

### Reagents

*Pyrobest*® DNA Polymerase and restriction enzymes *Nde*I and *Xho*I were purchased from TaKaRa Company (Japan). PCR purification kit, gel extraction kit, and plasmid miniprep kit were obtained from the Promega Company (USA). mPEG20 kDa-butyraldehyde (Alk-mPEG) and mPEG20 kDa-maleimide (Mal-mPEG) were purchased from Sigma–Aldrich (St. Louis, MO, USA). Heparin-Sepharose column, Sepharose G25 column and AKTA purifier were purchased from GE healthcare (Piscataway, NJ, USA). Enhanced chemiluminescence (ECL) detection kit was from Pierce (Rockford, IL, USA). Anti-Erk1/Erk2 rabbit mAb, anti-phospho-Erk1/Erk2 (Thr202/Tyr204) rabbit mAb were purchased from Cell Signaling Technology (Danvers, MA, USA). KGF(FGF-7)-basic Mini ELISA Development Kit was purchased from PeproTech (Princeton, NJ, USA). The protein assay reagent used for quantitative protein analysis was purchased from Bio-Rad (Hercules, CA, USA). Other reagents, unless otherwise indicated, were of the highest quality commercially available.

### Expression and Purification of rhKGF-1

RhKGF-1 was expressed as a fusion protein with bacterial glutathione-S-transferase (GST) at the N-terminus as described previously [43]. Briefly, the recombinant plasmid, pET-GST-KGF-1 was transformed into *Escherichia coli* BL21 (DE3) cells, then BL21 (DE3) cells were incubated at 37°C in Luria-Bertani (LB) medium containing ampicillin (50 µg/mL) until cell density reached an OD_600_ of 0.6. The cells were then transferred to 30°C, and 0.4 M isopropyl-1-thio-β-d-galactopyranoside (IPTG, Gold BioTechnology, St. Louis, MO) was added to the medium to induce expression of the recombinant product. After incubation for 3–4 h, the bacteria were collected by centrifugation and the cell pellet was then resuspended in 25 mM Tris-HCl (pH 7.2), 1 mM EDTA, 0.6–0.9 M NaCl, and 1 mM phenylmethylsulfonyl fluoride (PMSF), and then the cells were lysed by sonication and clarified by centrifugation. Soluble recombinant product was recovered from bacterial lysates by Glutathione Sepharose chromatography. Then, the GST and a portion of the N-terminal sequence was removed by incubation of the immobilized fusion product with 5 µg/mL thrombin in phosphate buffer (PB, pH 7.0) for 24 h at room temperature. The incubation mixture was applied to a CM Sepharose Fast Flow column (1 mL bed volume) pre-equilibrated with 15 column volumes (CVs) of binding buffer (20 mM sodium phosphate buffer, SPB, pH 7.0) at a rate of 0.5 mL/min. The sample was washed with 10 CVs of binding buffer, and then eluted with elution buffer A (0.3 M NaCl, 20 mM SPB, pH 7.0) , and B (1.0 M NaCl, 20 mM SPB, pH 7.0) over 10 CVs. The final product was further purified using Heparin Sepharose column with a PB elution buffer (pH 7.0) containing 0.6 M NaCl. All elution fractions were collected and analyzed by 12% SDS-PAGE, and then the rhKGF-1 fractions were stored at −80°C for the subsequent experiments.

### Solution-Phase PEGylation of rhKGF-1

PEGylation of rhKGF-1 with Alk-mPEG was performed at room temperature in a sodium phosphate buffer (20 mM, pH 6.0) in the presence of 20 mM NaBH_3_CN. Alk-mPEG was allowed to react with the protein at various PEG-to-protein molar ratios (5, 10, 15, and 20) for different periods of time. To determine the optimal conditions for the Mal-mPEG modification of rhKGF-1, the PEGylation was performed at room temperature in 20 mM sodium phosphate buffer at different PEG-to-protein ratios, pH, and reaction times. All reactions were terminated by adding 2% (w/v) glycine and then analyzed by 12% SDS -PAGE.

### Solid-Phase PEGylation of rhKGF-1

Three major procedures were performed for the solid-phase PEGylation of rhKGF-1: 1) Two mL of 0.1 mM rhKGF-1 in 20 mM sodium phosphate buffer (pH 6.0) was applied and bound on Heparin-Sepharose column. Due to the high binding affinity, rhKGF-1 completely bound on the column as evidenced by the fact that no protein was detected in the flow through after application of the protein sample on the column. 2) A solution containing 4.8 mL of 0.5 mM Alk-mPEG and 20 mM NaBH_3_CN was circulated through the column at low flow rate (0.01∼0.03 mL/min) for different periods of time (2, 4, 8 or 12 h). At the end, 30 mL of equilibrium buffer (20 mM sodium phosphate buffer, pH 6.0) was used to wash unreacted Alk-mPEG. 3) The reaction complex was completely eluted by applying 2 M NaCl in sodium phosphate buffer at a flow rate of 1 mL/min. The eluted fraction was collected and desalted by Sepharose G25 column and stored at −20°C. PEGylation yield was estimated by 12% SDS-PAGE and densitometry quantification as previously reported [19], [44].

### Purification of Solid-Phase PEGylated rhKGF-1

The reaction mixture obtained from the Sepharose G25 chromatography was applied on a SP Sepharose Fast Flow column (1 mL bed volume) pre-equilibrated with 15 column volumes (CVs) of binding buffer (20 mM sodium phosphate buffer, SPB, pH 7.0) at a flow rate of 0.5 mL/min. The sample was washed with 10 CVs of binding buffer, and then eluted with a salt gradient (from buffer A (0 M NaCl, 20 mM SPB, pH 7.0) to buffer B (1.0 M NaCl, 20 mM SPB, pH 7.0)). All elution fractions were collected and analyzed by 12% SDS-PAGE.

### Mass Spectrometry and N-Terminal Analysis of PEGylated rhKGF-1

Mass spectra were acquired using an Applied Biosystems Voyager System DE PRO MALDI-TOF mass spectrometer with a nitrogen laser. The matrix was a saturated solution of R-cyano-4-hydroxycinnamic acid in a 50/50 mixture of acetonitrile and water containing 0.1% trifluoroacetic acid. The mono-PEGylated protein and matrix were mixed at a ratio of 1/1, and then 1 µL sample was spotted onto a 100-well sample plate. All spectra were recorded on a Bruker Ultraflex instrument (Bruker Daltonik, Germany) and acquired in positive mode over the range 600–2500 Da under reflection conditions (20 kV accelerating voltage, 350 ns extraction delay time) and 2–100 kDa under linear conditions (25 kV accelerating voltage, 750 ns extraction delay time). The N-terminal amino acid sequence of PEGylated rhKGF-1 was examined by Edman degradation method [45], and followed by MALDI-TOF mass spectroscopy as described above.

### Analysis of the Mitogenic Activity of PEGylated rhKGF-1

We used NIH 3T3 cell line (American Type Culture Collection, Rockville, MD) to determine the proliferative effect of PEGylated rhKGF-1. First, the cells were seeded in 96-well plates with 5×10^3^ cells per well and incubated for 24 h. Then they were washed with phosphate buffered saline (PBS) and starved-cultured in the corresponding low glucose Dulbecco's Modified Eagle's Medium (DMEM) containing 0.4% Fetal Bovine Serum (FBS) for 24 h. The cells were then treated with 3 µM native rhKGF-1, 3 µM PEGylated rhKGF-1 for 24 h, and finally the number of viable cells was detected by the methylthiazoletetrazolium (MTT) method as previously reported [46], [47].

### Western Blot Analysis for the MAP Kinase Activation of PEGylated rhKGF-1

NIH 3T3 cells were treated with rhKGF-1 or PEGylated rhKGF-1 at three doses 1.5 µM, 3 µM, and 6 µM. After washing with PBS for three times, the cells were homogenized in a lysis buffer. Total cellular proteins were collected by centrifuging at 12,000 rpm at 4°C. The protein concentration was then determined by Bicinchoninic acid (BCA) kit. Next, the protein samples were mixed with loading buffer and subjected to electrophoresis on a 12% SDS-PAGE gel at 110 V for 100 min. Following transferring the proteins to a Polyvinylidine Fluoride (PVDF) membrane (Millipore-Billerica, MA, USA), the membrane was blocked with 5% nonfat milk for 2 hours at room temperature. The membrane was then washed three times with Tris-Buffered Saline containing 0.1% Tween-20 (TBST) and incubated with primary antibody at 4°C overnight. The membrane was then washed three times with TBST, and secondary HRP-conjugated antibody was applied and the membrane was incubated for 1 h at room temperature. Finally, immunoreactive protein bands were visualized by ECL system, and quantified by optical densitometer.

### Circular Dichroism Spectroscopy Analysis

The secondary structures of rhKGF-1 and PEGylated rhKGF-1 were determined with a circular dichroism (CD) spectropolarimeter (Model J-810, Jasco, Japan). Far-UV CD spectra were recorded at wavelengths between 190 and 250 nm using a 0.1 cm path length cell at 25°C with a protein concentration of 6 µM in 10 mM PBS, pH 7.0. Each spectrum was a representative of three scans. The CD spectra were corrected for buffer contributions.

### Effect of Solid-Phase PEGylation on the *In Vitro* Stability of rhKGF-1

Trypsin from Promega, Inc. (Madison, WI, U.S.A.) was dissolved at 100 mg/mL in 50 mM ammonium bicarbonate buffer (pH 7.8). PEGylated and non-PEGylated rhKGF-1 were treated with trypsin at a molar ratio of 10/1 (protein/trypsin) and 37°C for different periods of time as indicated and then 50 µL of 20% TFA was added to stop the digestion reaction. The samples were then subjected to SDS-PAGE to examine the protein integrity.

Samples for storage stabilities tests contained 1.0 mg/mL non-PEGylated and PEGylated rhKGF-1 prepared in 20 mM SPB (pH 7.0). These samples (1 mL each) were aliquoted into 3 mL type 1 glass vials in a sterile laminar flow hood. The vials were sealed with rubber stoppers (1888 Teflon, West Co.) and 13 mm flip-off aluminum seals were crimped in place. The vials were placed in the incubators set at 37°C for 3 days, then some of the samples were transferred to another incubators set at 45°C. After incubation for different periods of time as indicated, 100 µL of the samples were withdrawn and centrifuged at 4200 rpm. The supernatant of each sample was collected and finally the soluble protein concentration was determined using Bicinchoninic acid (BCA) kit.

To determine the pH stability of PEGylated and non-PEGylated rhKGF-1, the protein samples prepared in different buffers with various pH (4–5, 25 mM acetate; pH 6–7, 25 mM Hepes) were incubated for 30 min at room temperature. Following incubation, the protein samples were diluted 5000 times by bioassay medium and than used for mitogenic bioassay. The *in vitro* bioactivities of proteins were then measured by mitogenic bioassay as described above.

### Animals and Surgical Procedures

Normal male SD rats (220–250 g) were used based on the procedures approved by the Animals Care and Use Committee from Jilin University [license No. SYXK(Ji)2010-0007]. The animal production license is No. SCXK 2010-0003.

### Pharmacokinetic Evaluation of Solid-Phase PEGylated rhKGF-1 in Rats

Following intravenous (i.v.) injection of the intact and PEGylated proteins at a dose of 100 µg/kg body weight (six animals in each group), blood samples were obtained at time points indicated. Serum levels of the test proteins were quantified using KGF(FGF-7)-basic Mini ELISA Development Kit (PeproTech, USA; sensitivity range of 63 to 4000 pg/mL). Pharmacokinetic parameters of the test proteins were determined by Drug and Statistics Software (DAS, Version 2.0; Mathematical Pharmacology Professional Committee of China). The elimination half-life (t_1/2_) was calculated using the formula t_1/2_ = 0.693/K_e_ (K_e_ stands for elimination rate constant).

### Evaluation of Hepatoprotective Effect of Solid-Phase PEGylated rhKGF-1 Using a Rat Model of Acute Liver Failure

48 male SD rats were randomly divided into four groups (12 rats/group): enalpril interventional group A (rhKGF-1-pretreated groups), enalpril interventional group B (PEGylated rhKGF-1-pretreated groups), injury-model group and healthy control group. Rats in interventional groups were injected intraperitoneally with either rhKGF-1 or PEGylated rhKGF-1 with a dose of 0.1 µmol/kg. This optimized dose was obtained from our preliminary animal experiments. Rats in the injury-model and control groups were injected intraperitoneally with normal saline. After 24 hours, rats in the test and the injury-model groups were injected intraperitoneally with 2.5 mL/kg CCl_4_ (Kanto Chemistry, Tokyo, Japan) dissolved in olive oil (Kanto Chemistry, Tokyo, Japan), and rats in control group received normal saline injection again. 4 hour after intoxication, all rats were sacrificed and then the levels of AST and ALT were determined in their serum using full automatic biochemical analyzer as reported previously [48].

### Statistical Analysis of the Experimental Data

The *in vitro* experiments were performed three times with triplicate samples for each individual experiment. All data were expressed as mean ± SD and subjected to statistical analysis by ANOVA and student *t*-test using statistical software NASDAQ: SPSS from SPSS Inc.

## Supporting Information

Figure S1 **SDS-PAGE and CD analysis of Mal-PEGylated rhKGF-1.** Panel A. SDS-PAGE analysis of solution-phase Mal-PEGylation of rhKGF-1 in the absence or presence of 2 M urea. Lane M, molecular weight standards; lanes a and b correspond to the PEGylation reaction mixtures in the absence of 2 M urea; lanes c and d correspond to the PEGylation reaction mixtures in the presence of 2 M urea. Panel B. Far-UV CD spectra of non-PEGylated (black line), solid-phase PEGylated rhKGF-1 (red line) and solution-phase Mal-PEGylated rhKGF-1 (blue line). The ellipticities are reported as mean residue ellipticity (MRE) (deg cm2 dmol-1). Panel C. Ribbon presentation of KGF-1 structure. Amino (N) and carboxyl (C) terminal ends are indicated, the Cys 40 is also labeled.
(DOC)

Figure S2 **SDS-PAGE analysis of the solution-phase Alk-PEGylated rhKGF-1.** Lane M, molecular weight standards; lane a, non-PEGylated rhKGF-1; lane b, solid-phase PEGylated product achieved at reaction time of 8 h and PEG PEG-to-protein molar ratio of 10; lane c–f, PEGylation products obtained at reaction time of 8 h and PEG-to-protein molar ratio of 5, 10, 15 and 20, respectively.
(DOC)

Figure S3 **Elution profile of the solid-phase Alk-PEGylation mixture from Heparin-Sepharose column.**
(DOC)
