# Supplementary material for: A Novel Solid-Phase Site-Specific PEGylation Enhances the In Vitro and In Vivo Biostabilty of Recombinant Human Keratinocyte Growth Factor 1
Source: PLoS One. 2012 May 4;7(5):e36423. doi: 10.1371/journal.pone.0036423 (PMC3344868; doi:10.1371/journal.pone.0036423)
Supplement: Figure S1 — SDS-PAGE and CD analysis of Mal-PEGylated rhKGF-1. Panel A. SDS-PAGE analysis of solution-phase Mal-PEGylation of rhKGF-1 in the absence or presence of 2 M urea. Lane M, molecular weight standards; lanes a and b correspond to the PEGylation reaction mixtures in the absence of 2 M urea; lanes c and d correspond to the PEGylation reaction mixtures in the presence of 2 M urea. Panel B. Far-UV CD spectra of non-PEGylated (black line), solid-phase PEGylated rhKGF-1 (red line) and solution-phase Mal-PEGylated rhKGF-1 (blue line). The ellipticities are reported as mean residue ellipticity (MRE) (deg cm2 dmol-1). Panel C. Ribbon presentation of KGF-1 structure. Amino (N) and carboxyl (C) terminal ends are indicated, the Cys 40 is also labeled. (DOC) [file pone.0036423.s001.doc]

**
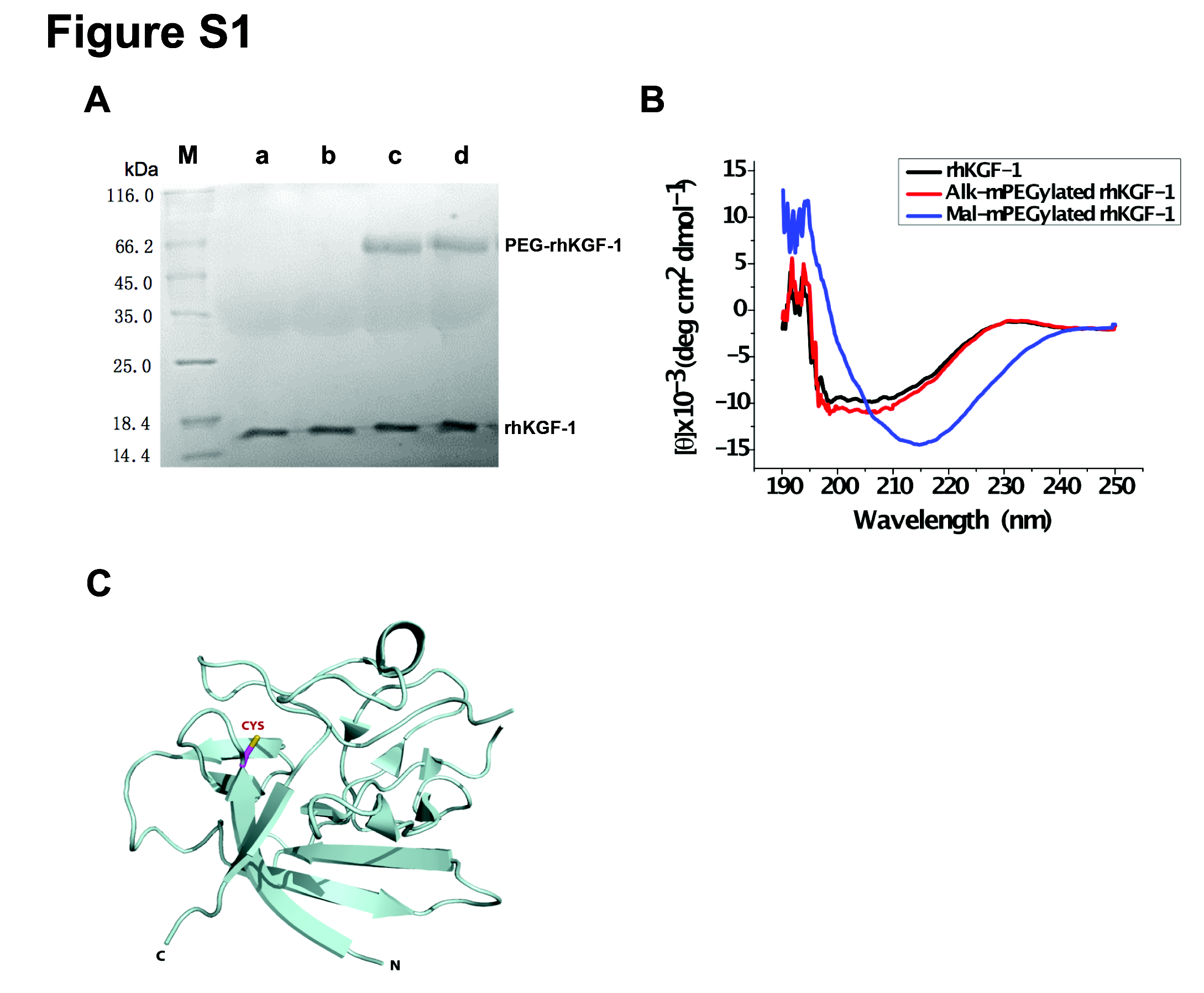
**

**Figure S1. SDS-PAGE and CD analysis of Mal-PEGylated rhKGF-1.** Panel A. SDS-PAGE analysis of solution-phase Mal-PEGylation of rhKGF-1 in the absence or presence of 2 M urea. Lane M, molecular weight standards; lanes a and b correspond to the PEGylation reaction mixtures in the absence of 2 M urea; lanes c and d correspond to the PEGylation reaction mixtures in the presence of 2 M urea. Panel B. Far-UV CD spectra of non-PEGylated (black line), solid-phase PEGylated rhKGF-1 (red line) and solution-phase Mal-PEGylated rhKGF-1 (blue line). The ellipticities are reported as mean residue ellipticity (MRE) (deg cm2 dmol-1). Panel C. Ribbon presentation of KGF-1 structure. Amino (N) and carboxyl (C) terminal ends are indicated, the Cys 40 is also labeled.
