# Supplementary material for: A Novel Solid-Phase Site-Specific PEGylation Enhances the In Vitro and In Vivo Biostabilty of Recombinant Human Keratinocyte Growth Factor 1
Source: PLoS One. 2012 May 4;7(5):e36423. doi: 10.1371/journal.pone.0036423 (PMC3344868; doi:10.1371/journal.pone.0036423)
Supplement: Figure S2 — SDS-PAGE analysis of the solution-phase Alk-PEGylated rhKGF-1. Lane M, molecular weight standards; lane a, non-PEGylated rhKGF-1; lane b, solid-phase PEGylated product achieved at reaction time of 8 h and PEG PEG-to-protein molar ratio of 10; lane c–f, PEGylation products obtained at reaction time of 8 h and PEG-to-protein molar ratio of 5, 10, 15 and 20, respectively. (DOC) [file pone.0036423.s002.doc]

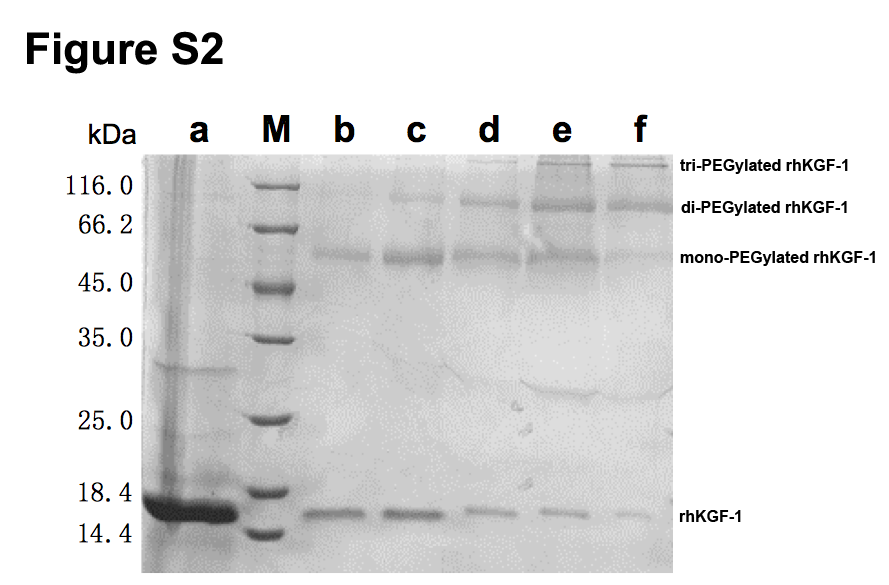


**Figure S2. SDS-PAGE analysis of the solution-phase Alk-PEGylated rhKGF-1.** Lane M, molecular weight standards; lane a, non-PEGylated rhKGF-1; lane b, solid-phase PEGylated product achieved at reaction time of 8 h and PEG PEG-to-protein molar ratio of 10; lane c-f, PEGylation products obtained at reaction time of 8 h and PEG-to-protein molar ratio of 5, 10, 15 and 20, respectively.
