# Supplementary material for: A Novel Solid-Phase Site-Specific PEGylation Enhances the In Vitro and In Vivo Biostabilty of Recombinant Human Keratinocyte Growth Factor 1
Source: PLoS One. 2012 May 4;7(5):e36423. doi: 10.1371/journal.pone.0036423 (PMC3344868; doi:10.1371/journal.pone.0036423)
Supplement: Figure S3 — Elution profile of the solid-phase Alk-PEGylation mixture from Heparin-Sepharose column. (DOC) [file pone.0036423.s003.doc]

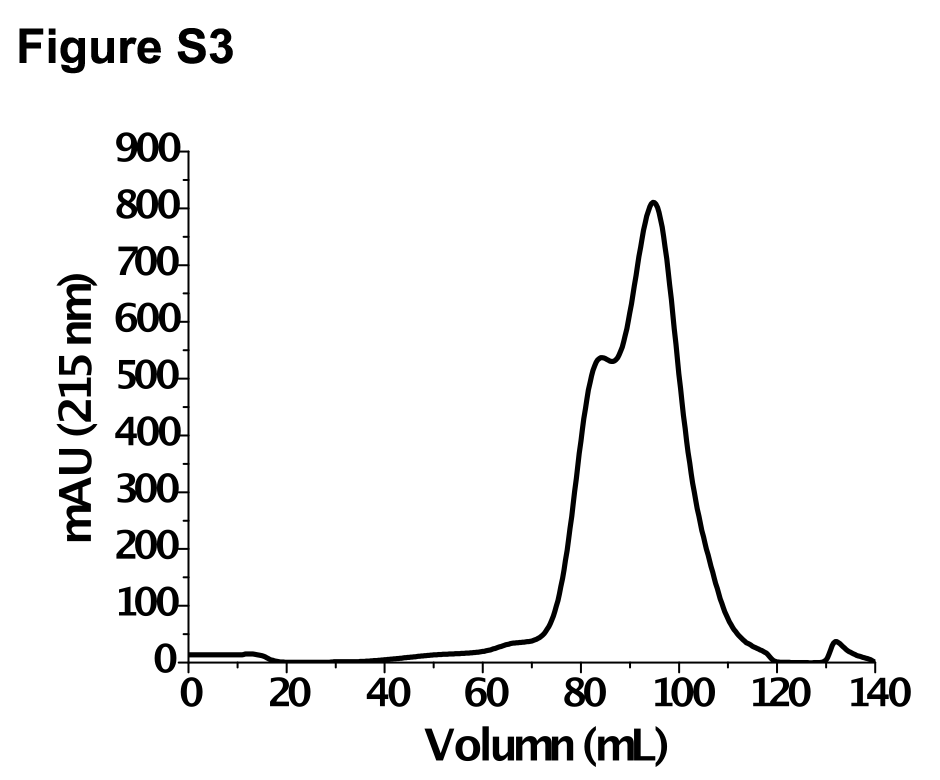


**Figure S3. Elution profile of the solid-phase Alk-PEGylation mixture from Heparin-Sepharose column**.
